# Items

**Published:** 1882-07

**Authors:** 


### Items.

IODOFORM INSANITY. — Schede has noticed that the use of iodoform has been attended with marked psychical symptoms. One type which is very noticeable among children is marked by dullness of the special senses, vomiting, and spasms of single groups of muscles. In adults, Schede has on two occasions seen general mental confusion, loss of personal identity, loud singing and violence. He has had under observation cases of melancholia attonita, also two cases of melancholia with frenzy, and three cases of simple melancholia; all arising from the use of iodoform.

THE NATURE OF INSANITY.—Dr. Charles Mercier (*Journal of Mental Science*, January, 1882) discusses the nature of insanity, and endorses the opinion that insanity was the failure of adjustment of the organism to its environment, expressed by Hughlings Jackson before his paper was completed. Exactly the same opinion was expressed in an article on insanity in *Gaillard's Medical Journal*, Vol. II, p. 452, in slightly different terms.—*Journal of Nervous and Mental Disease.*

Mr. F. M. Balfour was on Wednesday, May 31, elected to the new professorship of Morphology in the University of Cambridge. Prof. Balfour is a young man, having taken his degree, through the Natural Sciences Tripos, in 1873; and it is to be hoped that the duties of his professorship will not interfere with his continuance of the work of original investigation for which he has shown himself so eminently qualified.

There is no such tonic as happiness.

